# Enhancing clinical decision support with genomic tools in breast cancer: A Scottish perspective

**DOI:** 10.1016/j.breast.2024.103728

**Published:** 2024-04-13

**Authors:** A.L. Peters, P.S. Hall, L.B. Jordan, F.Y. Soh, L. Hannington, S. Makaranka, G. Urquhart, M. Vallet, D. Cartwright, H. Marashi, B. Elsberger

**Affiliations:** a Beatson West of Scotland Cancer Centre, Gartnavel Hospital, NHS Greater Glasgow & Clyde, 1053 Great Western Rd, Glasgow G12 0YN, UK; b Cancer Research UK (CRUK) Scotland Institute, Switchback Road, Bearsden, Glasgow G61 1BD, UK; c Edinburgh Cancer Research Centre, University of Edinburgh, Western General Hospital, Crewe Road South, Edinburgh, EH4 2XR, UK; d Ninewells Hospital & Medical School, NHS Tayside, Department of Pathology, Dundee, DD1 9SY, UK; e Raigmore Hospital, NHS Highland, Department of Oncology, Inverness IV2 3UJ, UK; f Aberdeen Royal Infirmary, NHS Grampian, Department of Breast Surgery, Aberdeen AB25 2ZN, UK; g Aberdeen Royal Infirmary, NHS Grampian, Department of Oncology, Aberdeen AB25 2ZN, UK

**Keywords:** Breast cancer, genomic test, Recurrence score, Chemotherapy, Progesterone receptor

## Abstract

**Introduction:**

The *Oncotype DX Breast RS* test has been adopted in Scotland and has been the subject of a large population-based study by a Scottish Consensus Group to assess the uptake of the recurrence score (RS), evaluate co-variates associated with the RS and to analyse the effect it may have had on clinical practice.

Materials & Methods: Pan-Scotland study between August 2018–August 2021 evaluating 833 patients who had a RS test performed as part of their diagnostic pathway. Data was extracted retrospectively from electronic records and analysis conducted to describe change in chemotherapy administration (by direct comparison with conventional risk assessment tools), and univariate/multivariate analysis to assess relationship between covariates and the RS.

**Results:**

Chemotherapy treatment was strongly influenced by the RS (p < 0.001). Only 30 % of patients received chemotherapy treatment in the intermediate and high risk PREDICT groups, where chemotherapy is considered. Additionally, 55.5 % of patients with a high risk PREDICT had a low RS and did not receive chemotherapy. There were 17 % of patients with a low risk PREDICT but high RS who received chemotherapy.

Multivariate regression analysis showed the progesterone receptor Allred score (PR score) to be a strong independent predictor of the RS, with a negative PR score being associated with high RS (OR 4.49, p < 0.001). Increasing grade was also associated with high RS (OR 3.81, p < 0.001). Classic lobular pathology was associated with a low RS in comparison to other tumour pathology (p < 0.01). Nodal disease was associated with a lower RS (p = 0.012) on univariate analysis, with menopausal status (p = 0.43) not influencing the RS on univariate or multivariate analysis.

**Conclusions:**

Genomic assays offer the potential for risk-stratified decision making regarding the use of chemotherapy. They can help reduce unnecessary chemotherapy treatment and identify a subgroup of patients with more adverse genomic tumour biology. A recent publication by Health Improvement Scotland (HIS) has updated guidance on use of the RS test for NHS Scotland. It suggests to limit its use to the intermediate risk PREDICT group. Our study shows the impact of the RS test in the low and high risk PREDICT groups. The implementation across Scotland has resulted in a notable shift in practice, leading to a significant reduction in chemotherapy administration in the setting of high risk PREDICT scores returning low risk RS. There has also been utility for the test in the low risk PREDICT group to detect a small subgroup with a high RS.

We have found the PR score to have a strong independent association with high risk RS. This finding was not evaluated by the key RS test papers, and the potential prognostic information provided by the PR score as a surrogate biomarker is an outstanding question that requires more research to validate.

## Highlights

• Real-world data across NHS Scotland, providing insights into the implementation of genomic decision tools in the management of EBC
• The RS test has caused significant decreases in chemotherapy use with benefits including reduced patient morbidity and cost savings for the NHS.
• The PR score is a strong independent predictor of the RS with future integrated use with genomic tests an avenue of future research and validation.

## 1 Introduction

Personalised decision making is essential in the management of early breast cancer (EBC). This will lead to improved outcomes for patients, and facilitate an individualised cancer journey. Improved knowledge of tumour heterogeneity and differentially expressed genes has led to the development of genomic signature assays.

There are various validated genomic tests currently available, with initial evidence from the retrospective analysis of the *TransATAC* database [1]. These include the Oncotype DX® Breast Recurrence Score® (RS) assay (Genomic Health Inc., Redwood City, CA), MammaPrint® (Agendia BV, Amsterdam, The Netherlands), Prosigna (PAM50 assay, Veracyte, San Francisco, CA) and EndoPredict (EPclin, Myriad Genetics, Zurich, Switzerland) [[1], [2], [3], [4], [5], [6], [7], [8]]. The RS test, according to numerous guidelines has prognostic validity [[9], [10], [11], [12], [13], [14], [15], [16], [17], [18], [19], [20], [21]]. It has been approved for use in hormone positive breast cancer in patients with node negative or up to three lymph nodes positive disease across Scotland, and is included in the ESMO Early Breast Cancer Guidelines [9,22].

The objective of this pan-Scotland study was to assess its use as a decision-making tool, and to investigate patient and tumour factors that may be associated with the RS. An additional objective was to describe the RS test's concordance with conventional decision tools such as PREDICT.

## 2 Background

There has always been uncertainty regarding which patients with EBC will benefit from adjuvant chemotherapy. It is clear that patients with HER2 positive or triple negative breast cancer benefit from systemic chemotherapy [[23], [24], [25]]. Evidence for substantial benefit from chemotherapy in patients with HR positive, HER2 negative EBC is less robust.

Due to this uncertainty and the need to try to reduce treatment side effects, several risk assessment tools have been developed over the years. The first of these was the *Nottingham Prognostic Index* (NPI) [25]. The NPI groups patients into a high risk (>5.4), moderate risk (3.4–5.4) or low risk (<3.4) group considering factors such as tumour size, nodal involvement and tumour grade.

Following on from NPI, Adjuvant Online! and then the PREDICT Breast NHS tool (PREDICT) were developed using national cancer registry data, and utilise clinicopathological factors from the patient and their tumour to estimate individual benefit from chemotherapy [26]. PREDICT is the decision tool most commonly in use in Scotland, and is available open access online allowing use in the routine clinical setting. PREDICT has been validated in a Scottish general EBC population [27].

Genomic tests will complement conventional decision tools as they provide independent additional prognostic information. The RS test has been validated for this purpose by *TAILORx* [10], and more recently in the node positive setting by *RxPONDER* [11]. It has also been proposed that the RS test may predict which patients benefit more from chemotherapy.

The National Institute for Health and Care Excellence Diagnostic Guidance 10/34 (NICE DG10/34) publications assessed current evidence for its use as a prognostic and predictive tool, its clinical utility, decision impact and also its cost effectiveness [9]. The prognostic ability of the RS test has been displayed clearly by several studies, with a high score being independently associated with a higher risk of recurrence and hence larger absolute benefit from chemotherapy [[10], [11], [12], [13], [14], [15], [16]].

The prognostic utility of the RS test was also shown across multiple studies (e.g. *WSG-PlanB*), displaying the correlation with increasing score and poorer prognosis [[17], [18], [19], [20]]. Whilst a low or high RS is clearly able to differentiate between patients with a high risk of recurrence and a low risk of recurrence, it is less clear for scores between 18 and 30 where the risk of recurrence is less certain. This led to further assessment in the TAILORx study.

*TAILORx* was a large prospective trial that assessed the clinical validity of the RS test [10]. Patients had a RS test performed on their tumour sample post operatively and were split into 3 groups. Group 1 (RS 0–10) received endocrine therapy alone. Group 2 (RS 11–25) received endocrine therapy or chemoendocrine therapy by randomisation. Group 3 (RS > 25) were registered to receive chemoendocrine therapy but were not randomised. The trial assessed both the predictive and prognostic ability of the RS test, concluding that for patients <50 years of age chemotherapy likely would have benefit in the intermediate score [11-25] range. For patients >50 years of age, a score of 25 or less indicated that chemotherapy is not required. TAILORx therefore provided a crucial insight into the cut offs for low or high risk of recurrence with regards to the RS test, and showed that it was likely to be age dependent.

The decision impact of the RS test was also shown across a number of UK and EU based studies which compared post-test chemotherapy decisions with a hypothetical pre-test decision [[18], [19], [20], [21], [22], [23], [24], [25], [26], [28],[29], [30], [31], [32], [33], [34]]. A change in treatment was found across all of these studies, suggesting an impact of genomic testing as a decision tool in determining chemotherapy administration. Additionally, cost effectiveness was displayed across many studies, subject to a range of test price discounts [[35], [36], [37], [38], [39], [40], [41], [42], [43], [44], [45]].

*RxPONDER* has recently been published as a follow-on trial after *TAILORx* in the node positive setting. Retrospective analysis from the *SWOG 8814* trial had suggested a predictive utility in the node positive (1–3 nodes) setting for the RS test [14,15]. The *RxPONDER* trial randomised patients with a low RS test (0-25) to endocrine or chemoendocrine therapy. The initial interim analysis found that patients with a low recurrence score in the postmenopausal group could safely avoid chemotherapy, but that an intermediate [11-25] score group exists for pre-menopausal patients where chemoendocrine therapy is still indicated. The effects of ovarian function suppression (OFS) were not directly assessed and may have been a confounding factor. There were 16 % of patients in the endocrine arm that had OFS, but only 3 % in the chemoendocrine arm [11].

Based on a review of the evidence behind the RS test, Health Improvement Scotland (HIS) recommended its use throughout NHS Scotland and have included in an update its use in patients with up to 3 positive lymph nodes. The updated recommendation also suggests that the RS test is used for patients who have intermediate risk scores either from NPI or from PREDICT, and that the test is unlikely to derive additional benefit for patients in the low risk or high risk setting [46]. Since the initial recommendation, there have been several large real world retrospective studies that have investigated the RS test.

A large study by L. M. McSorley et al. assessing the experience of the RS test across Ireland was published recently [29]. They included 963 patients between October 2011 and February 2019 who were node negative and hormone receptor positive, and had the RS test, were analysed. They found that 62.5 % had a change in treatment due to the RS test and were switched to endocrine therapy only. Cost analysis was performed with a predicted estimate of 1.9 million Euros saved and 73 % of patients avoiding chemotherapy.

There have been two retrospective studies by C. Markopoulos et al., published in 2016 and then in 2019 with updated data [45,47]. They looked at the health economics of using genomic decision tools and showed a possible relationship between the steroid receptor status and the RS test.

## 3 Materials/Methods

Patients throughout NHS Scotland who received an RS test as part of their decision-making regarding adjuvant therapy for EBC were included. We retrospectively collected all 833 consecutive patients between August 2018–August 2021. The patient list was accessed via the company *Exact Science,* which provides the RS test for NHS Scotland. This was the only involvement of industry in the study.

Patient demographics (Table 1) were collected along with details of their treatment decisions and their tumour pathology. The data was collated anonymously on *Excel* by a group of collaborating clinicians, located in the different health boards covering all of Scotland, agreeing on variables to be included prior to any data collection to unify the data and avoid missing data points. After successful Caldicott approval and completion of the data collection locally, data was anonymised and collated in an overall national master database for analysis. The database was analysed on *R* v4.1.0 with descriptive statistics and logistic regression being performed.

**Table 1:** Patient demographics by treatment received.

		Treatment Received		
Variable	N	Chemotherapy, N = 248	Endocrine Alone, N = 585	p-value
**Age**	833	53 [46-61]	57 [49-64]	**<0.001**
**Presentation**	833			0.30
Screening/Other		128 [28]	325 (72)	
Symptomatic		120 [32]	260 (68)	
**Menopausal status**	833			0.067
Postmenopausal		152 [28]	397 (72)	
Premenopausal		96 [34]	188 (66)	
**Charlson Comorbidity Index Score**	833	1.00 (0.00–2.00)	2.00 (1.00–3.00)	**0.014**
**RS Value**	833	31 [27-40]	15 [11-20]	**<0.001**
**Recurrence Score**	833			**<0.001**
<25		37 (6.3)	546 (94)	
>25		212 (85)	38 [15]	
**Predict Score (%)**	833	4.72 (3.50–6.00)	3.80 (2.90–4.71)	**<0.001**
**Predict Score**	833			**<0.001**
<3 (Low risk)		33 [17]	164 (83)	
3–5 (Intermediate risk)		115 [28]	298 (72)	
>5 (High risk)		100 [45]	123 [55]	
**NPI score**	833	4.44 (4.30–4.60)	4.30 (3.70–4.48)	**<0.001**
**Tumour Pathology**	833			**<0.001**
Classic Lobular		12 [10]	107 (90)	
Invasive Ductal		223 [36]	397 [64]	
Other		9 [17]	45 (83)	
Pleomorphic Lobular		4 [10]	36 (90)	
**Grade (Vertical Proportionality)**	833			**<0.001**
1		0 (0)	4 (0.7)	
2		48 (19.4)	296 (50.6)	
3		200 (80.6)	285 (48.7)	
**Tumour size(mm)**	833	23 [18-30]	24 [18-34]	0.14
**Multi-focal**	833	30 [20]	120 (80)	**0.004**
**LVSI**	833	95 [37]	163 [63]	**0.003**
**Nodal Stage**	833			0.36
N0		216 [30]	497 (70)	
N1		16 [33]	33 (67)	
N1mic		16 [23]	55 (77)	
**LN Pathology**	132			0.20
ITC		1 (8.3)	11 (92)	
Macrometastasis		16 [33]	33 (67)	
Micrometastasis		16 [23]	55 (77)	
**ER score**	833			**<0.001**
Moderately Positive		30 [56]	24 [44]	
Strong Positive		218 [28]	561 (72)	
**PR score**	833			**<0.001**
Moderately Positive		98 [41]	140 [59]	
Negative		68 [59]	47 [41]	
Strong Positive		77 [16]	395 (84)	
Unknown		5 [62]	3 [38]	
**Type of breast surgery**	833			0.28
Breast Conserving Surgery		187 [31]	420 (69)	
Mastectomy		61 [27]	165 (73)	
**Type of axillary surgery**	833			0.15
ANC		8 [44]	10 [56]	
N/A		0 (0)	2 (100)	
SLNB		239 [29]	573 (71)	
Unknown		1 (100)	0 (0)	

^1^Median (IQR); n (%).

^2^Wilcoxon rank sum test; Pearson's Chi-squared test; Fisher's exact test.

Tables were created using the gtsummary package on R, with p values calculated using either Pearson's Chi-squared, Fisher's exact or Wilcoxon rank sum tests depending on distribution and size of the variables. The Charlson Comorbidity Index was calculated, excluding the index breast cancer diagnosis, using an online calculator (https://www.mdcalc.com/calc/3917/charlson-comorbidity-index-cci). Boxplots show univariate assessment of the RS and covariates, with either a T Test, Wilcoxon rank sum or an ANOVA test used to calculate p values depending on distribution of the variable and number of groups involved.

PREDICT scores were calculated using the NHS online tool (https://breast.predict.nhs.uk/) based on each patient's clinicopathological parameters. The PREDICT scores were split into risk categories <3 % (low risk), 3–5% (intermediate risk) and >5 % (high risk) absolute benefit from chemotherapy at 10 years.

To further investigate the relationship between variables and the RS we constructed a multivariate logistic regression model. The outcome variable was RS and was split into either </=25 or >25. We constructed this model for the whole cohort, and also for the low risk (<3 %) PREDICT group to investigate further the small number of high risk RS patients.

## 4 Results

### 4.1 Demographics

Patient demographics split into treatment categories are available on Table 1, with statistically significant results indicated in bold. A total of 833 patients were analysed with 70 % of patients having endocrine therapy alone, and 30 % having chemotherapy in addition to endocrine therapy. There were 76 % of patients with a PREDICT Breast score of >3 % estimated 10 year mortality benefit from chemotherapy, for whom chemotherapy would be routinely considered. The median age and IQR in the endocrine therapy alone group was higher at 57 [49-64] vs 53 [46-61] in the chemoendocrine group (p < 0.001). The Charlson Comorbidity Index Score was higher in the endocrine group also, reflective of an older patient group (p < 0.05). Most of the patients were node negative, but we have a small node positive cohort of 120 patients included for analysis.

Steroid endocrine receptor status was classified as per Allred criteria into strong positive [7-8], moderately positive [3-6] and negative (0–2). The RS test provided the results of the ER and PR single gene scores. For clarity, any use of the terms ER score or PR score in this study pertain to the Allred score provided by routine pathology. The single gene scores will be referenced as the ER or PR single gene score. In this population of early breast cancer, 6.5 % of patients were ER score moderately positive. In contrast, 29 % of patients had PR score moderately positive and 15 % were negative.

### 4.2 PREDICT breast NHS score

With regards to PREDICT Breast, 23.6 % of patients returned a score of <3 %. Of this low risk group normally not offered chemotherapy it was found that 17 % received, due to a high RS.

Additional to this finding, 27 % of patients returned a PREDICT Breast score >5 %. It is expected to discuss chemotherapy in this high risk group (unless contraindicated due to patient comorbidities or otherwise), however 55.2 % were given endocrine therapy alone (p < 0.001).

Across the >3 % group where chemotherapy is discussed, only 33.8 % of this group had chemotherapy (p < 0.001). This contrasts with the intermediate to high risk RS groups where 73 % of patients had chemotherapy. Only 5 % of patients with a low risk RS had chemotherapy (p < 0.001).

**Fig. 1:**
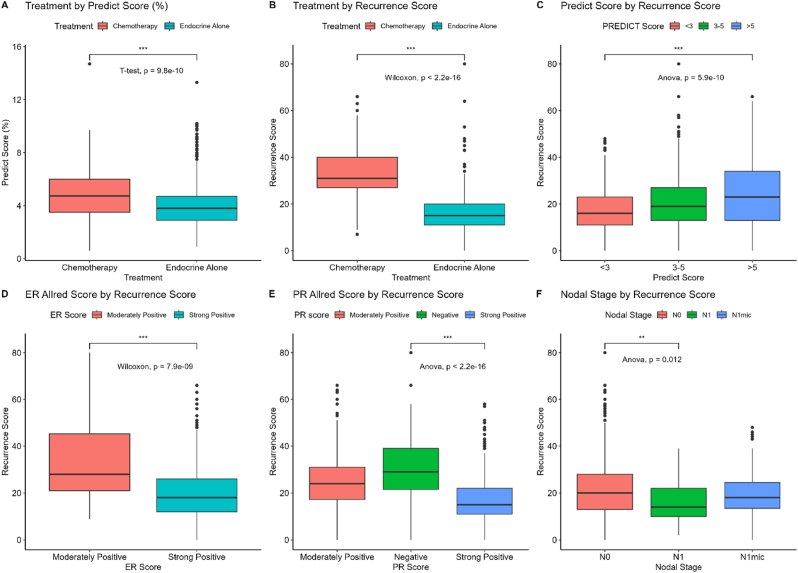
Boxplots A and B show relationship between Predict and Recurrence Score with Treatment. C-E show relationship between Predict score and ER/PR with Recurrence Score.Boxplot F shows relationship between nodal stage and Recurrence Score.

The relationship between PREDICT score, RS, treatment and the ER/PR score is shown in Fig. 1. Boxplots A and B show the influence both scores have on treatment, with the RS having a much stronger relationship (p < 0.001). Whilst *Boxplot C* does show that higher RS is associated with high risk PREDICT score >5 %, there is not a clear separation and the distribution of the low risk <3 % group does cross into high RS values.

The relationship with the ER/PR score and the RS is shown on *Boxplots D and E.* Moderately positive ER/PR score and negative PR score is associated with a high RS (p < 0.001).

**Fig. 2:**
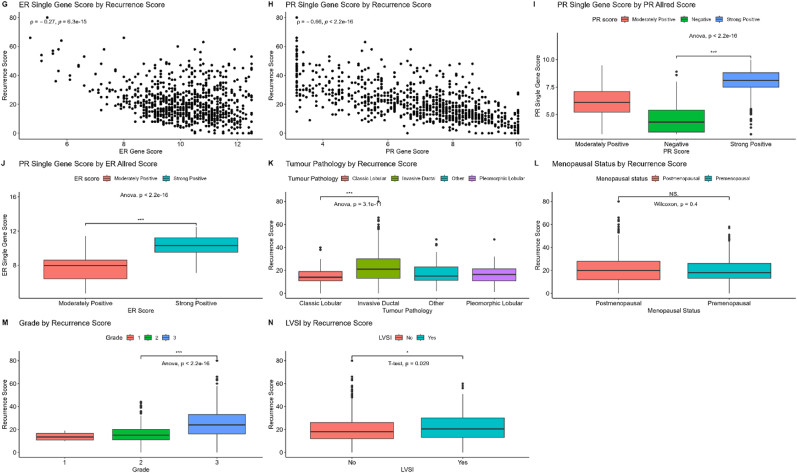
Scatter Plots showing relationship between Single Gene Scores and Recurrence Score with Spearmans' correlation coefficient. Boxplots I and J show relationship between the Allred score and the single gene scores. Boxplot K–N shows relationship between other covariates and Recurrence Score.

Fig. 2 displays the *Scatterplots G and H*, showing the relationship between the single gene scores and the RS. Whilst both are inversely proportional to the RS, the PR single gene score is the strongest predictor (Spearman's correlation coefficient ρ = -0.66, p < 0.001). Boxplots I and J show that the ER/PR scores are directly related to the ER/PR single gene scores.

The relationship between the other covariates and the RS are shown on Fig. 1, Fig. 2 (*Boxplots F & K-N)*. Nodal positivity and menopausal status did not seem to influence the RS. Tumour grade 3 and (to a lesser extent) LVSI were associated with high RS, but nevertheless 59 % of patients with grade 3 had endocrine therapy alone due to low RS.

### 4.3 Regression analysis

Table 2 contains the results from the multivariate logistic regression. The influence covariates had on the RS (</ = 25 versus >25) was analysed.

**Table 2:** Logistic Regression Analysis of Recurrence Score </=25 vs > 25.

	Univariate				Multivariate		
Characteristic	N	OR^a^	95 % CI^a^	p-value	OR^a^	95 % CI^a^	p-value
**PREDICT Score**	833						
<3		–	–		–	–	
3-5		2.40	1.55 to 3.81	**<0.001**	2.20	1.23 to 4.01	**0.009**
>5		4.71	2.96 to 7.67	**<0.001**	4.22	1.97 to 9.21	**<0.001**
**ER Score**	833						
Moderately Positive		–	–		–	–	
Strong Positive		0.29	0.16 to 0.51	**<0.001**	0.39	0.18 to 0.82	**0.014**
**PR Score**	825						
Moderately Positive		–	–		–	–	
Negative		2.78	1.76 to 4.46	**<0.001**	4.49	2.52 to 8.23	**<0.001**
Strong Positive		0.26	0.18 to 0.37	**<0.001**	0.26	0.17 to 0.40	**<0.001**
**Grade**	833	7.35	5.01 to 11.1	**<0.001**	3.81	2.10 to 7.09	**<0.001**
**Tumour Size**	833						
<2 cm		–	–		–	–	
2–5 cm		0.81	0.60 to 1.11	0.20	0.92	0.57 to 1.48	0.73
>5 cm		0.29	0.12 to 0.60	**0.002**	0.43	0.12 to 1.46	0.18
**Nodal Stage**	833						
N0		–	–		–	–	
N1		0.56	0.26 to 1.11	0.11	0.68	0.25 to 1.73	0.43
N1mic		0.69	0.38 to 1.20	0.20	0.55	0.26 to 1.15	0.12
**LVSI**	833						
No		–	–		–	–	
Yes		1.66	1.21 to 2.27	**0.001**	1.35	0.90 to 2.04	0.15
**Menopausal status**	833						
Postmenopausal		–	–		–	–	
Premenopausal		0.83	0.60 to 1.14	0.25	1.11	0.73 to 1.68	0.63
**Tumour Pathology**	833						
Classic Lobular		–	–		–	–	
Invasive Ductal		13.6	6.07 to 39.0	**<0.001**	6.55	2.40 to 21.7	**<0.001**
Other		3.97	1.26 to 13.7	**0.021**	2.36	0.61 to 9.78	0.22
Pleomorphic Lobular		3.26	0.86 to 12.3	0.074	1.42	0.33 to 6.09	0.63

a OR = Odds Ratio, CI = Confidence Interval.

The factors associated with a low RS were PREDICT <3 %, strong positive ER/PR and classic lobular pathology.

Factors associated with a high risk RS were negative PR score OR 4.49 (2.52–8.23, p < 0.001) and increasing Grade OR 3.81 (2.10–7.09, p < 0.001). Nodal stage and menopausal status did not show an association with the RS.

Separate regression analysis on Table 3 was performed in the <3 % low risk PREDICT group to assess for factors associated with a high RS (found in 17 %).

**Table 3:** Logistic Regression Analysis of Recurrence Score </=25 vs > 25 in low risk PREDICT <3 %.

	Univariate				Multivariate		
Characteristic	N	OR^a^	95 % CI^a^	p-value	OR^a^	95 % CI^a^	p-value
**ER Score**	197						
Moderately Positive		–	–		–	–	
Strong Positive		0.35	0.11 to 1.38	0.10	0.48	0.10 to 2.57	0.37
**PR Score**	195						
Moderately Positive		–	–		–	–	
Negative		9.81	3.42 to 30.6	**<0.001**	9.73	3.14 to 33.4	**<0.001**
Strong Positive		0.22	0.06 to 0.72	**0.017**	0.25	0.06 to 0.86	**0.034**
**Grade**	197	4.21	1.88 to 9.59	**<0.001**	1.67	0.45 to 7.34	0.47
**Tumour Size**	197						
<2 cm		–	–		–	–	
2–5 cm		0.39	0.16 to 0.86	**0.023**	0.44	0.11 to 1.79	0.23
**LVSI**	197						
No		–	–		–	–	
Yes		1.26	0.49 to 2.98	0.61	1.66	0.53 to 5.00	0.37
**Menopausal status**	197						
Postmenopausal		–	–		–	–	
Premenopausal		0.42	0.15 to 1.04	0.077	0.82	0.23 to 2.74	0.75

a OR = Odds Ratio, CI = Confidence Interval.

The factor associated with a high risk RS in the <3 % group was a negative PR score OR 9.73 (3.14–33.4, p < 0.001). Again, menopausal status had no association. There were no patients with N1mic with a high RS, in the <3 % group, so interpretation in this group was difficult and therefore nodal stage was not included in the regression model.

The adoption of the RS test has resulted in a significant change in clinical practice, as evidenced by the difference in chemotherapy administration (30 %) compared to the >3 % PREDICT Breast NHS group (76 %), where chemotherapy would be considered. Among the >5 % high risk PREDICT Breast NHS group (27 %), traditionally associated with almost always receiving chemotherapy, endocrine therapy was administered 55.5 % of the time. This highlights the substantial clinical impact of the RS test, leading to a reduction in chemotherapy administration by over 50 % in the high risk PREDICT group.

## 5 Discussion

This study shows real world population data exhibiting the use of a genomic decision tool in EBC throughout NHS Scotland. The PR score plays a significant role in influencing the risk of recurrence, as demonstrated in this study. It is worth noting that PREDICT does not consider the PR score when calculating risk. In contrast, the RS test provides a more precise assessment through the ER and PR single gene scores. Notably, we found that the PR single gene score exhibits the strongest association in comparison to the ER single gene score.

Through multivariate regression, we have found the PR score to be a strong independent predictor of the RS with an inverse relationship. It has previously been shown to be an independent prognostic variable, with a negative PR score associated with poor prognosis [48]. We are not the first to report an association between the PR score and the RS [[49], [50], [51], [52], [53], [54], [55], [56], [57], [58], [59], [60]], however the key Oncotype trials did not look at the PR score and therefore this relationship has only been shown by real world data out with clinical trial settings. We have one of the larger datasets showing this association. Work by Pawloski K et al. developed a supervised machine learning algorithm with a high degree of accuracy for predicting the RS, with the PR score being the strongest independent predictor [61]. The PR score is potentially a cheap and ready for use surrogate marker of the risk of recurrence, and more research to validate this finding is one of the outstanding questions. The recent HIS publication recommending using the RS test only in the intermediate risk PREDICT group makes no mention of incorporating the PR score into this assessment.

Conventional risk assessment tools such as PREDICT and NPI rely on clinicopathological factors. Our study demonstrates a correlation between the PREDICT score and the RS, but this linkage is not strong. It appears that while conventional clinicopathological factors are related to the RS, there is a tendency to overestimate risk, as described earlier. Within the low risk subgroup, we identified a small subset where risk is underestimated, indicated by a high RS, leading to chemotherapy administration. These patients would not have routinely received chemotherapy. This study would support ongoing consideration to use genomic tests in the low and high risk PREDICT groups, especially when the PR score is not strongly positive. A combination approach offered by RSClin, EPclin or Prosignia would appear to be an effective means of assessing risk of recurrence. Whether these tests could be improved by incorporating the PR score is one of the questions this work raises.

The clinical uptake of the test, and its effect on the individualised management of breast cancer patients, has been assessed across all regions of Scotland. Since the publication of *TAILORx*, and NICE DG34/10, the RS test has been adopted throughout NHS Scotland in EBC. The RS test has led to a substantial decrease in chemotherapy administration when compared to PREDICT. This has profound multifactorial implications for service delivery and patient experience.

It is anticipated that genomic tools will increasingly be employed in Oncology across various tumour types. Estimating risk solely based on clinicopathological parameters, such as those used by PREDICT and NPI, is imperfect as they fail to capture the true heterogeneity of genomic and transcriptional expressions that drive tumorigenesis and metastasis. The RS test has demonstrated, particularly through a dedicated retrospective analysis of the TransATAC dataset, the provision of additional prognostic information not offered by current risk assessment tools [1]. As previously mentioned we suggest a combined genomic/clinicopathological approach to be the future of breast cancer recurrence risk assessment, and more research is required in this area to develop new tests and validate existing ones.

We assessed the relationship between clinicopathological parameters and the RS. Consistent with findings from the TAILORx study [10], we observed that some of these parameters do not correlate with the RS. Specifically, factors such as nodal staging and menopausal status did not demonstrate an association with a high RS in our small node positive group. Our findings align with the initial interim results of the RxPONDER study, which suggested that nodal staging may not influence the RS [11]. Clinical trials like POSNOC are investigating the existence of a low risk node positive group, which could potentially avoid local axillary management if other adjuvant therapies are administered [62].

We found that increasing tumour grade and the presence of lymphovascular space invasion (LVSI) were associated with higher RS, which is in line with expectations as grade reflects cancer cell activity, and the RS test report includes the Ki67 gene. Although PREDICT takes these factors into account, the lack of standardized Ki67 reporting across pathology departments often limits its availability. Perhaps future directions with machine read Ki67, digitalised pathology and potentially AI driven reporting could lead to further improvements. This is clearly another crucial area of future research.

Classic Lobular pathology was shown to be strongly linked to a low RS. It is important to highlight that this study differentiated between classical and pleomorphic lobular subtypes, the latter being known to be more aggressive in nature.

Considering the cost implications of the RS test for the NHS, there may be clinical scenarios where the test could potentially be avoided, and patients could proceed directly to endocrine therapy. For instance, in cases of classic lobular subtype with a strong PR Allred score. However, it is evident that the RS test provides cost-effective benefits by reducing unnecessary chemotherapy administration in many other patient scenarios, particularly the high risk PREDICT group.

Additionally, patients with a low risk PREDICT score should still be considered for the RS test as some patients exhibited a high RS nevertheless. These patients had a RS test mainly due to either an intermediate NPI score or due to the presence of nodal disease. There were 16 patients in our database that did not fit into any of these categories, so the reason for testing is unclear as out with DG34/10 approval and also the updated HIS recommendation; however 4 of these patients did have a high risk RS test. The risk in these seemingly lower-risk patients appeared to be driven by the PR score. This lends further support to the combined approach and the introduction of the forthcoming RSClin test is expected to further enhance treatment decision-making in EBC [63]. EPclin and Prosignia clearly also have a role to play in this setting, and more research to delineate the best test moving forward is required.

Further advancements in adjuvant therapy for EBC are anticipated. Targeted therapies like CDK4/6 inhibitors, already extensively used as first-line treatment in advanced hormone-positive breast cancer [64], and antibody-drug conjugates (ADCs) may reshape the adjuvant treatment landscape. Having precise risk assessment tools with robust validation will become increasingly necessary.

Another potential avenue for further research in the RS test landscape is its use in the neoadjuvant setting. Knowing the risk of recurrence preoperatively in the setting of large tumours, or large tumour to breast ratio could be advantageous as we could give downstaging chemotherapy in such cases with confidence that they would have required adjuvant chemotherapy anyway if there was a high risk RS test. Surgical planning for advanced reconstruction can be facilitated by the patient undergoing systemic therapy upfront leading to a more efficient patient cancer treatment journey.

The strengths of this study include the substantial patient population of over 800 patients and its pan-Scotland scope, which helped mitigate regional biases. Limitations of the study include its retrospective nature, absence of recurrence and survival data at present, limiting the analysis to logistic regression. Additionally, accurate recording of ovarian function suppression was not consistent across all regions, but this data will be valuable for future analysis as it matures.

## 6 Conclusion

In summary, the implementation of genomic assays in the management of EBC will facilitate personalised decision-making regarding adjuvant therapy by providing accurate information on individual tumour risk for recurrence and metastasis. These assays have the potential to reduce unnecessary chemotherapy administration, especially in the high risk PREDICT group. Additionally, they enable treatment intensification for a subgroup within the low risk PREDICT group who exhibit more adverse genomic tumour biology. The use of genomic decision tools ultimately improves patient outcomes and this study supports their continued use in low and high risk PREDICT settings. Patients with indolent disease can be spared the morbidity of chemotherapy, while those with higher-risk profiles can receive beneficial treatment. This personalised approach enhances overall patient care.

We have found the PR score to be a strong independent predictor of the RS and support other real world studies who have also shown this. This is an area of important future research, and potential incorporation into next generation combined clinical/genomic assays. Work to better stratify which patient groups should receive a RS test could be centred around the PR score on the basis of the strong real world evidence that has emerged.

Moreover, the reduction in chemotherapy observed in this study suggests cost savings for the NHS. This is particularly crucial given the budgetary challenges faced by healthcare systems in addressing the growing complexities of Oncology and other medical fields. A health economic analysis in Scotland is currently on the way.

## Ethical approval

As this was a retrospective observational study formal ethical approval was not required. However, this work was approved by the local Caldicott Guardian at all regional sites that contributed to the study. The Caldicott Guardian ensures that the information sharing process and handling of any anonymised patient data is upheld to the highest standards.

## CRediT authorship contribution statement

**A.L. Peters:** Conceptualisation, Data curation, Formal analysis, Methodology, Project administration, Writing – original draft, Writing – review & editing. **P.S. Hall:** Conceptualisation, Data curation, Formal analysis, Methodology, Project administration, Resources, Supervision, Visualization, Writing – original draft, Writing – review & editing. **L.B. Jordan:** Conceptualisation, Data curation, Supervision, Writing – original draft, Writing – review & editing, Formal analysis, Investigation, Methodology, Project administration, Resources. **F.Y. Soh:** Conceptualisation, Data curation, Methodology. **L. Hannington:** Data curation. **S. Makaranka:** Data curation. **G. Urquhart:** Conceptualisation, Data curation, Formal analysis, Supervision, Writing – original draft, Writing – review & editing, Methodology, Project administration. **M. Vallet:** Data curation. **D. Cartwright:** Data curation, Formal analysis. **H. Marashi:** Conceptualisation, Data curation, Formal analysis, Methodology, Project administration, Supervision, Writing – original draft, Writing – review & editing. **B. Elsberger:** Conceptualisation, Data curation, Formal analysis, Methodology, Project administration, Supervision, Writing – original draft, Writing – review & editing.

## Declaration of competing interest

Dr Adam L Peters has received a travel grant to attend the 10.13039/100014735Royal College of Radiology Clinical Oncology academic training day from the Beatson Cancer Charity.

Miss Beatrix Elsberger has had an advisor role with Exact Sciences, and has received a travel grant to attend a meeting in London. The funds received were put back into the departmental pot to help fund a research nurse.
